# MicroRNA-130b Suppresses Malignant Behaviours and Inhibits the Activation of the PI3K/Akt Signaling Pathway by Targeting MET in Pancreatic Cancer

**DOI:** 10.1007/s10528-024-10696-7

**Published:** 2024-04-12

**Authors:** Zilin Yang, Yuming Tang, Xuejiao Wu, Jiancheng Wang, Weiyan Yao

**Affiliations:** 1 Department of Gastroenterology, Ruijin Hospital, Shanghai Jiao Tong University School of Medicine, Shanghai, 200025 China; 2 Department of General Surgery, Ruijin Hospital Affiliated to Shanghai Jiao Tong University School of Medicine, Shanghai, 200025 China

**Keywords:** MicroRNA-130b, Pancreatic cancer, Bioinformatics, Disease progression, PI3K/Akt signaling pathway

## Abstract

There has been interested in the microRNAs’ roles in pancreatic cancer (PC) cell biology, particularly in regulating pathways related to tumorigenesis. The study aimed to explore the hub miRNAs in PC and underlying mechanisms by bioinformatics and fundamental experiments. RNA datasets collected from the Gene Expression Omnibus were analysed to find out differentially expressed RNAs (DERNAs). The miRNA-mRNA and protein–protein interaction (PPI) networks were built. The clinicopathological features and expressions of hub miRNAs and hub mRNAs were explored. Dual-luciferase reporter gene assay was performed to assess the interaction between microRNA and target gene. RT-qPCR and western blot were employed to explore RNA expression. The roles of RNA were detected by CCK-8 test, wound healing, transwell, and flow cytometry experiment. We verified 40 DEmiRNAs and 1613 DEmRNAs, then detected a total of 69 final functional mRNAs (FmRNAs) and 23 DEmiRNAs. In the miRNA-mRNA networks, microRNA-130b (miR-130b) was the hub RNA with highest degrees. Clinical analysis revealed that miR-130b was considerably lower expressed in cancerous tissues than in healthy ones, and patients with higher-expressed miR-130b had a better prognosis. Mechanically, miR-130b directly targeted MET in PC cells. Cell functional experiments verified that miR-130b suppressed cell proliferation, migration, promoted apoptosis, and inhibited the PI3K/Akt pathway by targeting MET in PC cells. Our findings illustrated the specific molecular mechanism of miR-130b regulating PC progress. The miR-130b/MET axis may be an alternative target in the therapeutic intervention of PC and provide an opportunity to deepen our understanding of the pathogenesis of PC.

**Supplementary Information:**

The online version contains supplementary material available at 10.1007/s10528-024-10696-7.

## Introduction

Pancreatic cancer (PC) is a kind of complex and deadly tumour in the digestive system, which is characterized by aggressive malignancy and dismal prognosis (Strobel et al. 2019). Although surgery, chemotherapy, and radiation therapy have made tremendous strides, the final prognosis of PC patients is still awful (Mizrahi et al. 2020). Thus, the molecular mechanisms of the tumorigenesis, progression, and metastasis in PC need to be discovered in depth to find new prognostic biomarkers and explore effective treatment methods for PC.

MicroRNA (miRNA) is a class of non-coding small RNAs, ranging in size from 20 to 24 nucleotides that participates in post-transcriptional gene regulation (Bi et al. 2020; Gu et al. 2022). By targeting messenger RNAs (mRNAs), miRNAs could regulate metabolic processes, tumourigenesis, cell invasion, metastasis, cell division, apoptosis, and other biological processes (Braga et al. 2021; Bi et al. 2020; Yan et al. 2020). These miRNAs can mediate the expression level of target genes, consequently influencing cancer occurrence. For instance, by directly acting on nuclear factor kappa B1, miR-497 inhibited the gemcitabine resistance, migration, and invasion abilities of PC stem cells (Yu et al. 2022); miR-4653-3p upregulation was linked to tumorigenesis and awful prognosis, partly by down-regulating HIPK2 in pancreatic ductal adenocarcinoma patients (Hirabayashi et al. 2022). By targeting PAK1, exosomal miR-485-3p suppressed cell metastasis in PC (Li et al. 2022). These differential genes and downstream target genes or pathways also provide new strategies and ideas for targeted therapy of PC. However, the involvement of these miRNAs in the tumorigenesis and development, particularly in PC, has not been fully defined.

Herein, to discover functional differential expressed RNAs, we analysed the RNA datasets from the Gene Expression Omnibus (GEO) database. Through bioinformatics methods and clinical validation, we found miR-130b was correlated with PC as a hub miRNA with highest degrees and downregulated in PC tissues. Subsequently, we further explored the correlation between miR-130b and its target hub gene as well as its downstream signaling, and verified the role and mechanism of miR-130b in PC, which provided a new insight for PC diagnosis and treatment. There is a pre-print that has previously been published (Yang et al. 2022).

## Materials and Methods

### Data Collection

Different RNA expression profiles between PC and non-cancerous (NC) tissues were obtained from the public database, GEO database (https://www.ncbi.nlm.nih.gov/geo/) (Barrett et al. 2013) according to the following terms: pancreatic AND (cancer OR carcinoma OR malignancy OR adenocarcinoma OR tumour) AND tissue. Both the “series” and “Homo sapiens” designations were examined. Other conditions included the following: (1) PC patients; (2) include both cancerous and non-cancerous tissue groups. Raw miRNA expression profiles were obtained from GSE24279 (Bauer et al. 2012) (136 PC tissues and 22 NC tissues), GSE32678 (Donahue et al. 2012) (25 PC tissues and 7 NC tissues), and GSE41369 (Frampton et al. 2014) (9 PC tissues and 9 NC tissues). Other three datasets, including GSE56560 (Haider et al. 2014) (28 PC tissues and 7 NC tissues), GSE60970 (Sandhu et al. 2015) (49 PC tissues and 12 NC tissues), and GSE62165 (Janky et al. 2016) (118 PC tissues and 13 NC tissues), were chosen to be as original mRNA datasets.

### Verification of Differentially Expressed RNAs (DERNAs)

The online GEO2R tool (http://www.ncbi.nlm.nih.gov/geo/geo2r/) (Diboun et al. 2006) was used to filter DERNAs between malignant tumours and normal samples in each dataset. The criteria for detecting DERNAs were established at *P* < 0.05 and | log2 (Fold Change, FC) |> 1.

### Gene Ontology (GO) Functional Annotation and Kyoto Encyclopedia of Genes and Genomes (KEGG) Pathway Enrichment Analyses

WEB-based GEne SeT AnaLysis Toolkit (Webgestalt, http://www.webgestalt.org/) was a powerful online tool to reveal gene functions. The functional annotations of GO and KEGG pathway analysis in target genes of DEmiRNAs were conducted using the Webgestalt database (Liao et al. 2019). Molecular function (MF), cellular component (CC), and biological process (BP) were established as GO annotation. *P* < 0.05 was defined as statistical significance for the enrichment.

### Construction of the miRNA–mRNA Networks

Potential interaction relationships between DEmiRNAs and DEmRNAs were discovered by online miRWalk2.0 tool (http://mirwalk.umm.uni-heidelberg.de/) (Sticht et al. 2018), containing both the Target Scan (Agarwal et al. 2015) database and miRDB (Y. Chen and Wang 2020) database. A score ≥ 0.95 was made as the cut-off value for predictive interaction analysis using miRWalk. The final functional mRNAs (FmRNAs) were identified by intersecting the target mRNAs of DEmiRNAs and DEmRNAs through bioinformatics. The RNA interaction co-regulation networks were visualized by Cytoscape software (version 3.6.1). *P* < 0.05 was deemed significant.

### Construction of the Protein–Protein Interaction (PPI) Network

In brief, FmRNAs were examined by the Search Tool for the Retrieval of Interacting Genes database (STRING, https://cn.string-db.org/), and then the interactions of FmRNAs were visualized by Cytoscape. The central nodes represented key miRNAs or genes with essential functions.

### Identification of Potential Hub RNAs

We estimated the degree value of RNA in the miRNA-mRNA and PPI networks to find the hub RNAs. miRNAs with degrees > 5 were selected as hub miRNAs in the miRNA-mRNA networks. We also recognized the FmRNAs with degree ≥ 2 as hub genes in the PPI network.

### Real-Time Quantitative Polymerase Chain Reaction (RT-qPCR)

The PC tissues and paired NC tissues were collected from 11 PC patients pathologically diagnosed between 2019 and 2021 at Gastroenterology Department, Ruijin Hospital, Shanghai Jiao Tong University. None of the patients underwent radiation or chemotherapy before surgical resection of the tumours.

Total RNAs were extracted by TRIzol Reagent (Invitrogen, Carlsbad, CA). miR-130b mimic or inhibitor, and MET siRNA purchased from Beijing TSINGKE Co., Ltd. (Beijing, China) was applied for overexpression or downregulation of miR-130b. TaqMan miRNA probes (Applied Biosystems, Foster City, CA) and the primers of target genes were used to detect miR-130b expression according to the manufacturer’s protocol and were measured by LightCycler® 480 II real-time PCR system software and QuantStudio™ 3 System. Expression profiles were normalized to U6 snRNA or GAPDH. The sequences of above probes were listed in Online Resource 1.

### Clinical Feature Analysis of FmRNAs in the PPI Network

FmRNAs were studied and visualized by the Gene Expression Profiling Interactive Analysis database (GEPIA, http://gepia.cancer-pku.cn/detail.php) (Tang et al. 2017), which integrated the data from The Cancer Genome Atlas (TCGA) database and Genotype Tissue Expression (GTEx) database. The FmRNAs were uploaded to GEPIA and linkedOmics (http://linkedomics.org/) (Vasaikar et al. 2018), which then performed the expression and associated survival analysis of the FmRNAs on clinical samples. | Log_2_FC |> 1 and* P* < 0.05 were regarded as significant.

### Cell Lines

The AsPC-1 cells and MIA PaCa-2 cells were PC cell lines bought from the Shanghai Institute of Cell Biology (Shanghai, China). These two cells were cultured in Dulbecco’s modified Eagle’s medium (DMEM, Gibco, Life Technologies, Carlsbad, CA, USA) joined with 10% FBS (Genial Biological Inc, Colorado, USA) and 1% double antibiotic (penicillin–streptomycin, Gibco) and incubated in a humidified incubator at 37 °C with 5% CO_2_.

### Cell Transfection

PC cells were incubated in plates for 20 h and then transfected with Lipofectamine 2000 (Invitrogen). The treatments were separated into five groups: the group transfected with miR-130b mimic, the group transfected with miR-130b inhibitor, the group transfected with si-MET, the group co-transfected with miR-130b inhibitor and si-MET, and the group transfected with control. Cells, which were placed on 6-well plates, were transfected with 100 pmol miR-130b mimic or 100 pmol miR-130b inhibitor to regulated the expression of miR-130b, or 100 pmol si-MET to inhibit the expression of MET. The cells were gathered at 48 h post-treatment for further experiments.

### Western Blotting

The total protein in the cells was extracted with radioimmunoprecipitation (RIPA) lysate premixed with phenylmethylsulfonyl fluoride (PMSF). After separating with 10% SDS-PAGE gel, transferring the isolated proteins on a PVDF membrane, and then blocked with 5% skim milk in TBST buffer. And then incubated separately in antibody at 4 °C overnight (Anti-MET Antibody, Bioss, 1:1000 dilution; Anti-TBL1XR1 Antibody, Bioss, 1:1000 dilution; Anti-p-Pi3k, CST, 1:1000 dilution; Anti-p-Akt Antibody, CST, 1:1000 dilution; Anti-GAPDH antibody, CST, 1:1000 dilution). The bands were washed 3 times with TBST solution for 10 min each time, and then incubated with the appropriate HRP-conjugated antibody (Abcam, 1:2000 dilution) on the shaker for 2 h at room temperature. Finally, the musical groups were cleansed using TBST solution strips on the shaker. The strips were then subjected to exposure in an exposure machine, utilizing the GBOX-chemi-XL1.4 from SYNGENE.

### Dual-Luciferase Reporter Gene Assays

The potential binding sequence of miR-130b in human MET mRNA was selected from the overlapping predicted targets in STARBASE and TargetScan. psiCHECK2-hMET-3’UTR-Mut and -WT, were synthesized by HarO Life, and lipofectamine 2000 was utilized to co-transfect luciferase reporter plasmid and miR-130b mimic/mimic-NC into AsPC-1 or MIA PaCa-2 cells. Cells were cultured at 37 °C, 5% CO_2_ for 2 days, and Dual-Luciferase Reporter System Kit (Meilunbio, Dalian, China) used to determine Renilla and firefly luciferase activities.

### Flow Cytometry

Apoptosis abilities were detected by an Annexin V-FITC/Propidium Iodide (PI) Apoptosis Detection Kit (Yeason, China) on the basis of the manufacturer’s protocol. The results were obtained using FlowJo software.

### Cell Proliferation Assay

3000–6000 cells were planted into each well of 96-well plates. Each received well came with a 10 μl cell counting kit-8 (CCK-8) reagent (Dojindo, Tokyo, Japan), and it was left to react for 2 h. The CCK-8 assays were performed at 0, 24, 48, and 72 h. Five replicates were tested for each group.

### Cell Wound-Healing Assay

After transfection, PC cells were inoculated in 6‐well plates and cultured in DMEM with 2% FBS. After the cells have grown to 70–90%, the cell monolayer was scratched slowly. A microscope (Olympus, Japan) was used to examine and photograph the linear wound at 0, 24, 48, and 72 h. The wound-healing rates were analysed by Image J software.

### Cell Migration Assays

The migration was also detected by Transwell Permeable Supports (6.5 mm Insert; Costar, Cambridge, Mass). After transfection, 3000–6000 cells were seeded in the upper chamber in DMEM without FBS, while the lower chambers were filled with DMEM with 20% FBS. After incubation at 37 °C with humidified 5% CO_2_ for 24 h, the cells were transferred through a filter, then the chambers were then treated according to the manufacturer's protocol. Stained cells were counted under a microscope in the whole field.

### Statistical Analysis

Data were expressed at the mean±SEM and statistical analysis was made by GraphPad Prism 7 software. To compare multiple groups, the one-way ANOVA was employed. Student's t- test was utilized for comparing between two groups. All experiments were carried out at least three times independently.

## Results

### Identification of DEmiRNAs and DEmRNAs in PC

First, multiple PC-associated RNA expression profiles were collected from the GEO database, as listed in Table 1. The total clinical characteristic data of six GEO datasets was shown in Online Resource 2. DEmiRNAs and DEmRNAs were identified by applying *P* < 0.05 and | log_2_FC |> 1. By setting the threshold above, 40 DEmiRNAs were detected to be significantly abnormal expressed in PC, including 31 up-regulated and nine down-regulated miRNAs, as presented in Table 2. Subsequently, a total of 1613 DEmRNAs were identified by adopting the same threshold, of which 1069 were up-regulated, and 544 were down-regulated, as listed in Online Resource 3. In addition, the top 5 DEmRNAs and DEmiRNAs were listed in Tables 3 and 4. Those DEmiRNAs and DEmRNAs were subjected to further analyses.

**Table 1:** Selected GEO datasets

Microarray	GEO accession	Number of tumour samples	Number of adjacent non-tumour samples
miRNA microarray	GSE24279	136	22
	GSE32678	25	7
	GSE41369	9	9
	Total	170	38
mRNA microarray	GSE56560	28	7
	GSE60979	49	12
	GSE62165	118	13
	Total	195	32

*GEO* Gene Expression Omnibus, *miRNA* microRNA, *mRNA* messenger RNA

**Table 2:** The 40 DEmiRNAs between pancreatic cancer samples and adjacent non-tumourous samples

		GSE24279		GSE32678		GSE41369	
miRNA ID	Up/down	*P*	Log_2_FC	*P*	Log_2_FC	*P*	Log_2_FC
hsa-let-7d	Up	–	–	0.021	1.570	0.016	1.354
hsa-let-7i	Up	< 0.001	1.308	–	–	0.001	2.073
hsa-miR-10a	Up	< 0.001	1.036	0.015	1.959	< 0.001	2.841
hsa-miR-125a-5p	Up	< 0.001	1.165	–	–	< 0.001	2.483
hsa-miR-125b	Up	< 0.001	1.325	–	–	0.002	2.846
hsa-miR-127-3p	Up	0.003	1.164	–	–	0.023	1.173
hsa-miR-130b	Down	< 0.001	− 3.917	–	–	0.001	− 2.612
hsa-miR-135b	Up	–	–	< 0.001	3.443	< 0.001	2.820
hsa-miR-141	Down	< 0.001	− 2.358	0.002	2.114	0.048	− 2.128
hsa-miR-145	Up	< 0.001	1.749	–	–	< 0.001	3.245
hsa-miR-150	Up	< 0.001	3.328	–	–	0.001	3.284
hsa-miR-151-5p	Up	–	–	0.033	1.339	0.008	1.534
hsa-miR-155	Up	< 0.001	3.580	0.002	− 1.457	0.002	1.327
hsa-miR-1825	Up	0.025	4.068	0.011	1.225	–	-
hsa-miR-184	Down	0.011	− 1.076	0.003	− 1.021	–	-
hsa-miR-196a	Up	–	–	0.038	1.113	0.004	3.586
hsa-miR-199a-3p	Up	< 0.001	1.226	0.046	1.782	0.001	2.445
hsa-miR-199a-5p	Up	< 0.001	1.689	–	–	< 0.001	3.264
hsa-miR-199b-3p	Up	< 0.001	1.222	0.046	1.782	–	-
hsa-miR-200c	Down	< 0.001	− 1.856	0.002	2.216	0.050	− 1.681
hsa-miR-21	Up	–	–	< 0.001	3.418	< 0.001	2.993
hsa-miR-210	Up	< 0.001	3.580	0.007	1.413	0.009	1.338
hsa-miR-216a	Down	< 0.001	− 4.743	–	–	0.001	− 2.636
hsa-miR-217	Down	< 0.001	− 5.425	–	–	0.002	− 3.572
hsa-miR-221	Up	< 0.001	1.803	0.028	1.278	< 0.001	2.182
hsa-miR-222	Up	< 0.001	2.396	0.001	2.004	0.001	1.572
hsa-miR-23a	Up	< 0.001	1.016	0.007	1.434	0.002	2.266
hsa-miR-24	Up	–	–	0.044	1.334	0.023	1.839
hsa-miR-27a	Up	–	–	0.040	1.617	< 0.001	3.636
hsa-miR-31	Up	< 0.001	3.444	0.007	2.315	0.001	1.615
hsa-miR-324-5p	Up	< 0.001	1.947	–	–	0.001	1.305
hsa-miR-331-3p	Up	< 0.001	1.060	–	–	< 0.001	1.831
hsa-miR-34a	Up	–	–	0.003	1.810	0.042	1.442
hsa-miR-34c-5p	Up	–	–	0.006	1.051	0.011	1.016
hsa-miR-425-3p	Down	0.001	− 1.054	0.005	− 1.112	–	-
hsa-miR-484	Up	0.001	1.136	–	–	< 0.001	1.836
hsa-miR-513b	Down	0.007	− 1.590	0.007	− 1.512	–	-
hsa-miR-523	Up	< 0.001	8.474	0.001	1.222	0.047	− 1.353
hsa-miR-526a	Down	–	–	0.003	− 1.278	0.009	− 1.487
hsa-miR-532-3p	Up	< 0.001	1.621	–	–	0.016	1.335

*DEmiRNA* differentially expressed miRNA

**Table 3:** The top 5 significantly up-regulated and top 5 down-regulated DEmiRNAs in three GEO datasets

miRNA name	Up/down	Pancreatic cancer (*n* = 170), normal pancreas (*n* = 38)					
		GSE24279		GSE32678		GSE41369	
		*P*	Log_2_FC	*P*	Log_2_FC	*P*	Log_2_FC
hsa-miR-217	Down	< 0.001	− 5.425	–	–	0.002	− 3.572
hsa-miR-216a	Down	< 0.001	− 4.743	–	–	0.001	− 2.636
hsa-miR-130b	Down	< 0.001	− 3.917	–	–	0.001	− 2.612
hsa-miR-141	Down	< 0.001	− 2.358	0.002	2.114	0.048	− 2.128
hsa-miR-200c	Down	< 0.001	− 1.856	0.002	2.216	0.050	− 1.681
hsa-miR-145	Up	< 0.001	1.749	–	–	< 0.001	3.245
hsa-miR-150	Up	< 0.001	3.328	–	–	0.001	3.284
hsa-miR-1825	Up	0.025	4.068	0.011	1.225	–	–
hsa-miR-135b	Up	–	–	< 0.001	3.443	< 0.001	2.820
hsa-miR-21	Up	–	–	< 0.001	3.418	< 0.001	2.993

*DEmiRNA* differentially expressed miRNA

**Table 4:** Top 5 significantly up-regulated and down-regulated DEmRNAs in three GEO datasets ( | Log_2_FC |> 3)

mRNA name	Up/down	Pancreatic cancer (*n* = 195), normal pancreas (*n* = 32)					
		GSE56560		GSE60980		GSE62165	
		*P*	Log_2_FC	*P*	Log_2_FC	*P*	Log_2_FC
ALB	Down	< 0.001	− 5.929	< 0.001	− 4.496	< 0.001	− 5.300
PNLIPRP1	Down	< 0.001	− 4.939	0.003	− 3.191	< 0.001	− 6.150
SERPINI2	Down	< 0.001	− 4.563	< 0.001	− 4.023	< 0.001	− 6.470
CELA2A	Down	0.001	− 4.272	0.009	− 3.827	< 0.001	− 6.020
AQP8	Down	< 0.001	− 3.681	< 0.001	− 4.068	< 0.001	− 6.170
MMP11	Up	< 0.001	1.952	< 0.001	3.881	< 0.001	4.860
GPRC5A	Up	< 0.001	3.513	< 0.001	2.296	< 0.001	4.320
SLC6A14	Up	< 0.001	3.930	< 0.001	2.996	< 0.001	4.650
POSTN	Up	< 0.001	4.379	< 0.001	3.026	–	–
CEACAM6	Up	< 0.001	4.549	< 0.001	3.920	< 0.001	3.960

*DEmRNA* differentially expressed mRNA

### Construction of the miRNA–mRNA Networks and the PPI Network

**Fig. 1:**
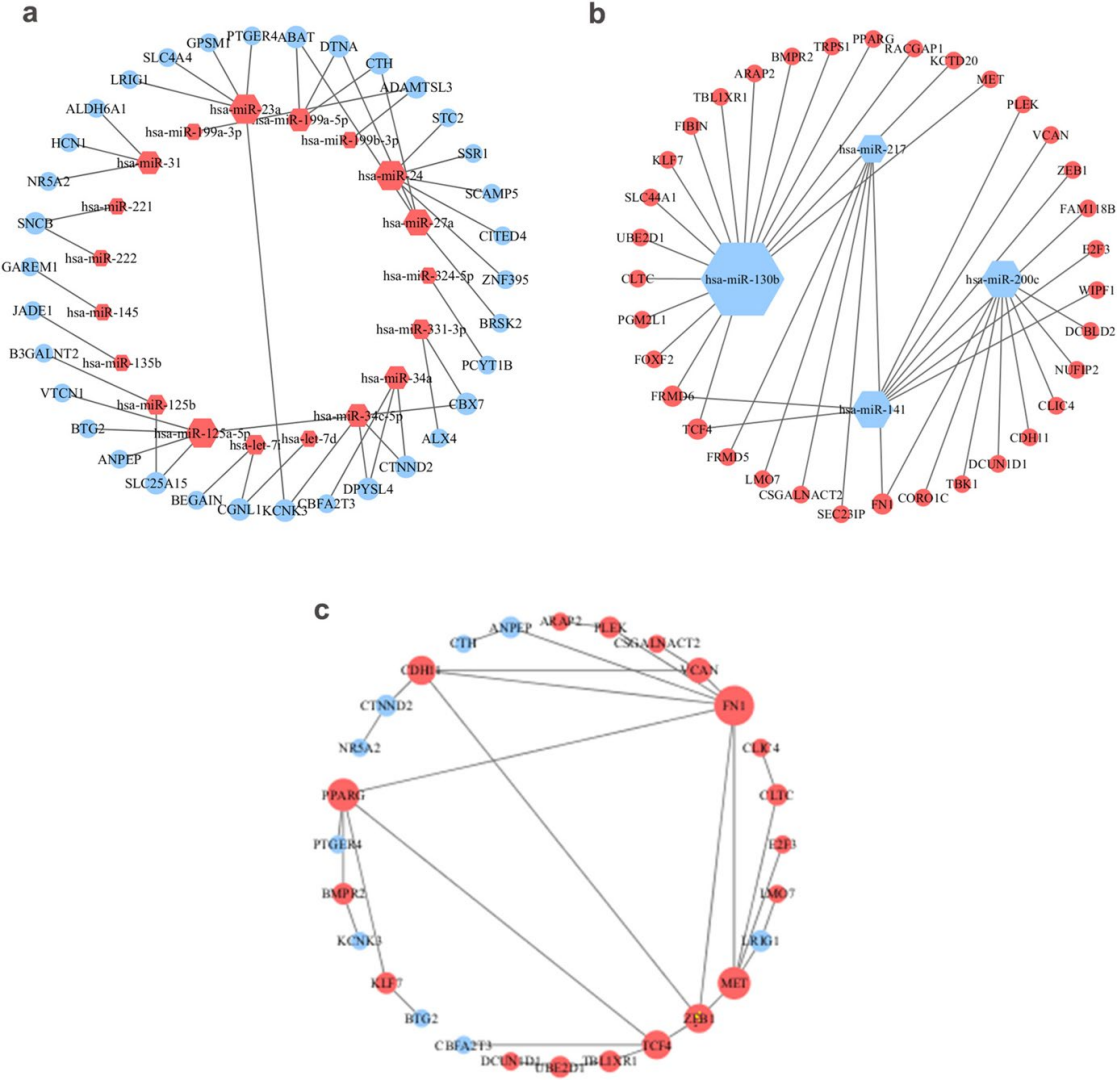
The miRNA-mRNA regulatory networks and PPI network. **a** The up-regulated DEmiRNAs-target genes regulatory network; **b** The down-regulated DEmiRNAs-target genes regulatory network. The hexagons and circles represented miRNAs and mRNAs, respectively. **c** protein–protein interaction network of the final functional mRNAs. Red represented the up-regulated RNA expression; blue represented the down-regulated RNA expression. *DE* differentially expressed, *miRNA* microRNA, *mRNA* messenger RNA

To reveal the molecular mechanism of PC, we used the miRWalk to predict the downstream genes of the 40 DEmiRNAs. The results indicated that a total of 1798 mRNAs targeted by 34 DEmiRNAs were obtained, comprising 2600 miRNA-mRNA pairs. When intersecting with DEmRNAs, 69 FmRNAs were identified. We used the overlapping DEmiRNAs and FmRNAs to build the miRNA-mRNA and PPI networks. The miRNA-mRNA networks were established containing 23 DEmiRNAs and 69 FmRNAs, which included 83 miRNA-mRNA axes, as shown in Online Resource 4 and Fig. 1a and b. In the miRNA-mRNA networks, 19 overexpressed DEmiRNAs down-regulated 34 FmRNAs, while four under-expressed DEmiRNAs up-regulated 35 FmRNAs. The PPI network contained 37 nodes and 36 edges, as illustrated in Fig. 1c.

### Identification of Hub RNAs

As listed in Table 5, hsa-miR-130b, hsa-miR-200c, hsa-miR-141, hsa-miR-24, hsa-miR-23a, hsa-miR-217, and hsa-miR-125a-5p were selected as hub miRNAs with degree ≥ 5, which reflected their essential roles in the miRNA-mRNA regulation networks. Among them, miR-130b got the highest degree score, so we took this miRNA for further investigation.

**Table 5:** Topology parameters of 7 hub miRNAs (degree ≥ 5) in the miRNA-mRNA networks

Gene	Type	Target mRNA
hsa-miR-130b	Down-regulated miRNA	TCF4, FRMD6, FOXF2, PGM2L1, CLTC, UBE2D1, SLC44A1, KLF7, FIBIN, TBL1XR1, ARAP2, BMPR2, TRPS1, PPARG, RACGAP1, KCTD20, MET
hsa-miR-200c	Down-regulated miRNA	DCBLD2, NUFIP2, CLIC4, CDH11, DCUN1D1, TBK1, CORO1C, FN1
hsa-miR-141	Down-regulated miRNA	PLEK, TCF4, VCAN, FRMD6, ZEB1, FAM118B, E2F3, WIPF1
hsa-miR-24	Up-regulated miRNA	STC2, SSR1, SCAMP5, CITED4, ZNF395
hsa-miR-23a	Up-regulated miRNA	LRIG1, KCNK3, SLC4A4, GPSM1, PTGER4
hsa-miR-217	Down-regulated miRNA	SEC23IP, CSGALNACT2, LMO7, FRMD5, FN1
hsa-miR-125a-5p	Up-regulated miRNA	SLC25A15, CBX7, ANPEP, BTG2, VTCN1

In addition, the PPI network showed that there were the top 10 hub mRNAs, including ALDH6A1, FN1, PPARG, MET, TCF4, ZEB1, CDH11, TBL1XR1, VCAN, and PLEK, in the PPI network, as presented in Table 6. MET, PPARG, TBL1XR1, and TCF4 were all the downstream target genes of miR-130b.

**Table 6:** The top 10 hub mRNAs in the PPI network

mRNAs	Type	Betweenness	Closeness	Degree
FN1	Up-regulated mRNA	0.552	0.458	7
PPARG	Up-regulated mRNA	0.381	0.415	5
MET	Up-regulated mRNA	0.336	0.391	5
TCF4	Up-regulated mRNA	0.279	0.375	4
ZEB1	Up-regulated mRNA	0.208	0.409	4
CDH11	Up-regulated mRNA	0.155	0.370	4
VCAN	Up-regulated mRNA	0.074	0.338	3
ALDH6A1	Down-regulated mRNA	1.000	1.000	2
TBL1XR1	Up-regulated mRNA	0.142	0.287	2
PLEK	Up-regulated mRNA	0.074	0.325	2

### Lowly Expressed miR-130b was Related to the Poor Prognosis of PC Patients

**Fig. 2:**
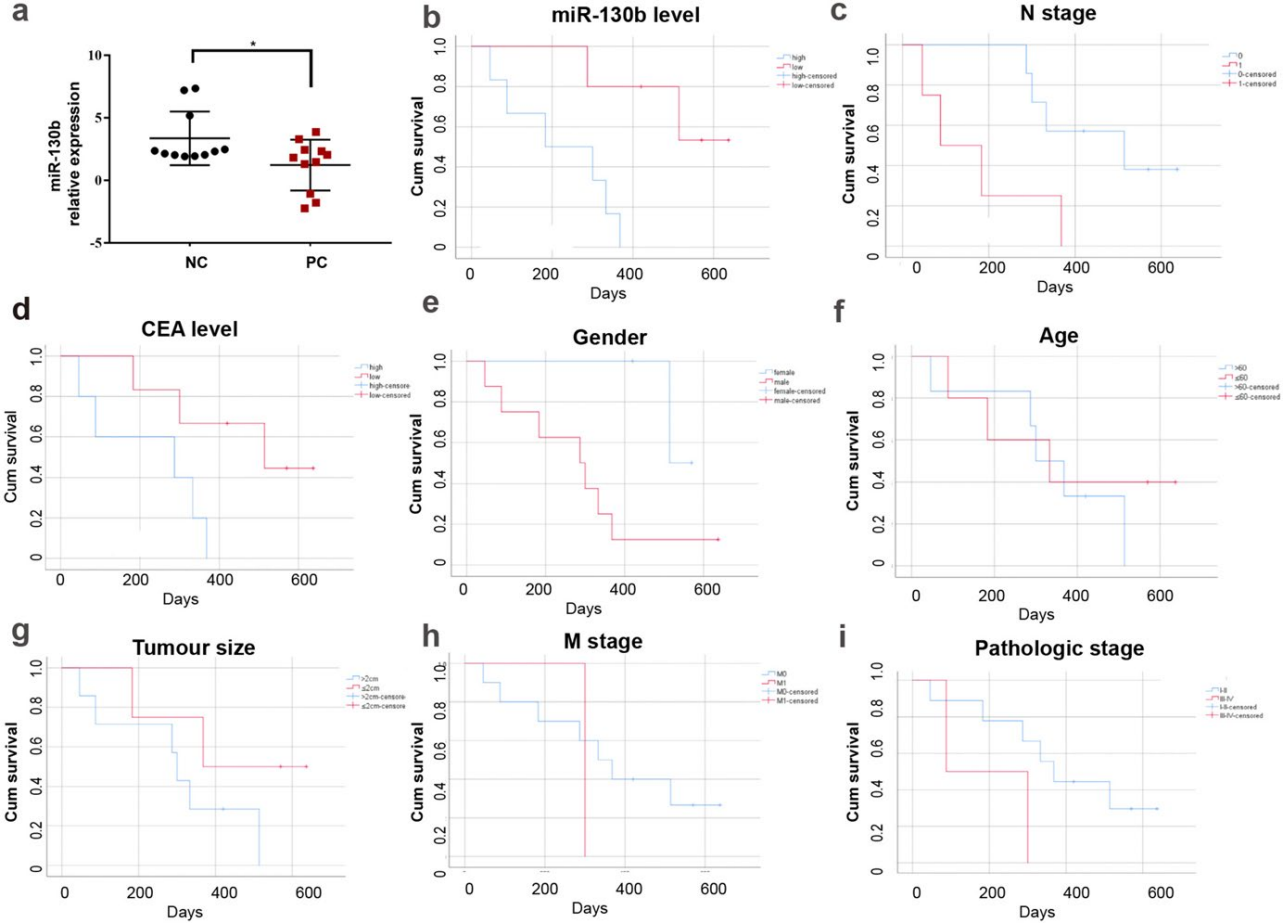
Expression of miR-130b in PC tissues and survival analysis of PC patients. **a** The relative expression of miR-130b in PC tissues (*n* = 11) and paired NC tissues (*n* = 11). **b** The survival analysis of patients according to miR-130b expression. **c** The survival analysis of PC patients according to N stage. **d** The survival analysis of PC patients according to CEA level. **e** The survival analysis of PC patients according to gender. **f** The survival analysis of PC patients according to age. **g** The survival analysis of PC patients according to tumour size. **h** The survival analysis of PC patients according to M stage. **i** The survival analysis of PC patients according to pathology stage. *PC* pancreatic cancer, *NC* non-cancerous, *CEA* carcinoembryonic antigen; *P < 0.05 was considered statistically significant

In the three miRNA datasets enrolled from the GEO database, the expression of miR-130b in the tumor subgroup of GSE24279 (*P* < 0.001) and GSE41369 (*P* < 0.001) was higher than that in the normal tissue subgroup, as listed in Table 3. Subsequently, we detected the expression of miR-130b in 11 PC tissues and the matched NC tissues by RT-qPCR assay, as shown in Fig. 2a and Table 7. miR-130b was downregulated in PC tissues compared with the non-carcinoma tissues (1.225±0.6129 vs 3.363±0.646, *P* = 0.0262). There were no correlation between miR-130b and clinical features of PC patients, including age, gender, tumour size, smoke, lymphatic metastasis, vital status, stage, vascular invasion, hypertension, diabetes, carcinoembryonic antigen (CEA) level, and carbohydrate antigen19-9 (CA19-9) level, as shown in Table 7.

**Table 7:** Relationship between clinical features of PC and the miR-130b content determined by RT-qPCR

Characteristics	*N*	*M*±SD	*P*
*Tissues*			
Adjacent non-cancerous tissues	11	3.363±0.646	0.026*
Pancreatic cancer	11	1.225±0.6129	
*Age (years)*			
≥ 60	6	0.827±2.109	0.879
< 60	5	1.558±1.713	
*Gender*			
Male	8	1.518±1.976	0.974
Female	3	0.445±1.590	
*Tumour size (cm)*			
≤ 3	8	3.068±0.764	0.507
> 3	3	0.534±1.787	
*Smoke*			
Yes	2	-1.651±0.578	0.674
No	9	1.864±1.507	
*Lymphatic metastasis*			
Yes	4	2.383±0.647	0.641
No	7	0.564±2.112	
*Vital status*			
Live	4	0.878±1.142	0.772
Death	7	1.424±2.247	
*Stage*			
Stage I–II	9	0.702±1.751	0.431
Stage III–IV	2	3.582±0.290	
*Vascular invasion*			
Yes	4	1.389±2.257	0.735
No	7	1.132±1.723	
*Hypertension*			
Yes	8	0.562±1.809	0.991
No	3	2.993±0.865	
*Diabetes*			
Yes	2	0.272±2.055	0.600
No	9	1.437±1.845	
*CEA level (ng/ml)*			
> 5	5	1.404±1.911	0.819
≤ 5	6	1.077±1.948	
*CA19-9 level (kU/l)*			
> 400	6	0.873±2.110	0.921
≤ 400	5	1.649±1.610	

*N* number, *SD* standard deviation, *M* mean

*Significant difference defined as *p* < 0.05

Survival analysis was employed to detect the effect of miR-130b as an indicator tool in PC prognosis. The Kaplan–Meier curve demonstrated the downregulation of miR-130b in 11 PC patients was associated with significantly shorter survival time (*P* = 0.014), as shown in Fig. 2b. The Kaplan–Meier survival curves in Fig. 2c, d showed that patients with lower N stage (*P* = 0.020) and higher CEA level (*P* = 0.034) had longer survival. On the other hand, there was no difference in the survival rate of patients with different ages, genders, T stages and M stages, etc., as shown in Fig. 2e–i.

### Function Enrichment Analysis of FmRNAs Targeted by miR-130b

**Fig. 3:**
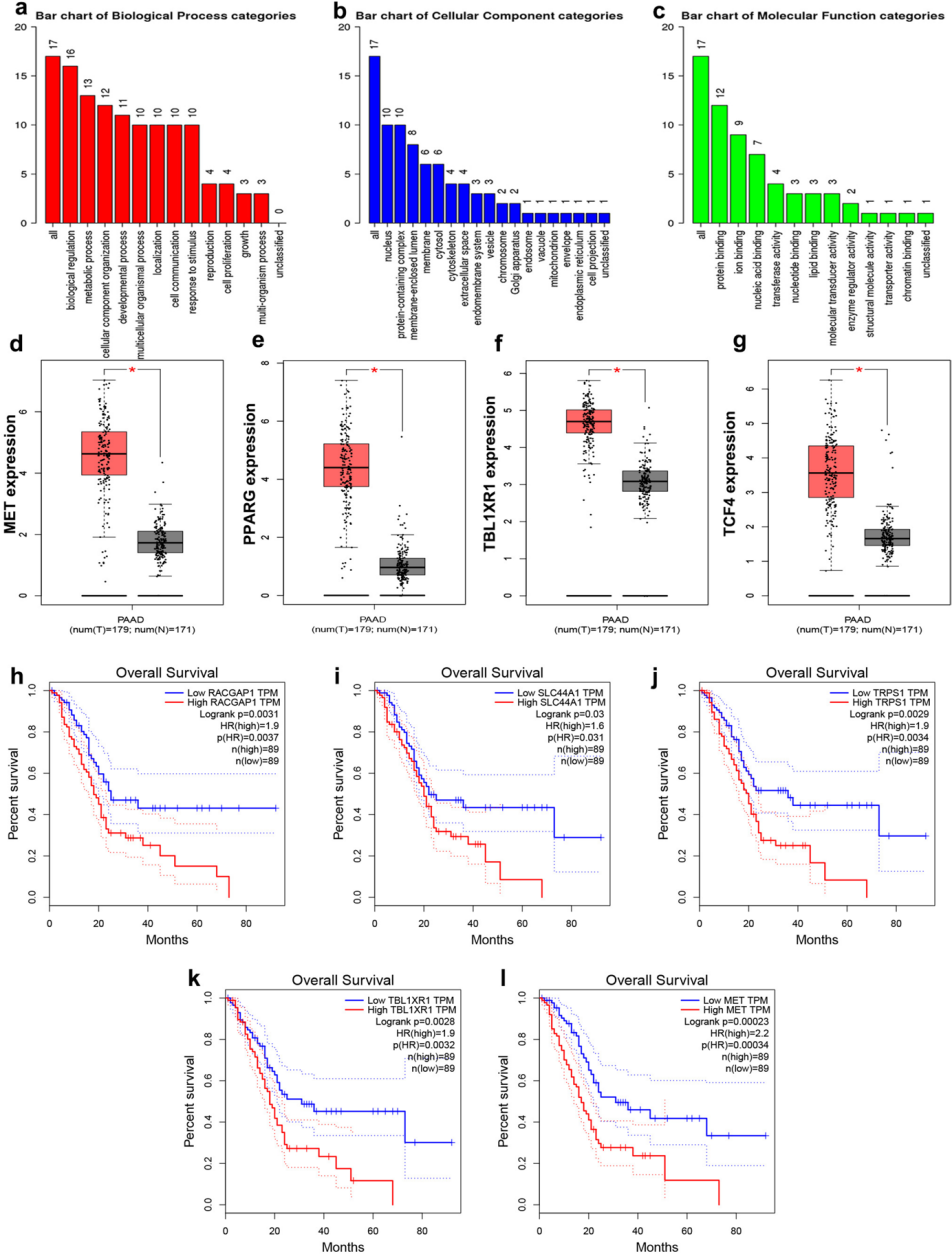
GO enrichment analysis, the expression and survival analysis of the FmRNAs targeted by miR-130b. **a–c** GO enrichment analysis of the FmRNAs targeted by miR-130b, including biological progress (**a**), cellular component (**b**), and molecular function (**c**) categories. **d–g** The expression of hub FmRNAs targeted by miR-130b, such as MET (**d**), PPARG (**e**), TBL1XR1 (**f**) and TCF4 (**g**), the red boxes represent the gene expression in PC (*n* = 179), and the grey boxes represent the gene expression in tissues of the normal pancreas tissues (*n* = 171); **h–l** The survival analysis of FmRNAs targeted by miR-130b originated from the GEPIA database in PC tissues. *GO* Gene Ontology, *FmRNAs* final functional mRNAs, *DEmRNA* differentially expressed mRNA. *P < 0.05 was considered statistically significant

In the above miRNA-mRNA networks, there were 17 FmRNAs targeted by miR-130b. These FmRNAs were analyzed by webgestalt for GO and KEGG analysis, as presented in Fig. 3a–c and Online Resource 5. The GO analysis showed that the downstream genes of miR-130b were linked to biological regulation, protein binding, and metabolic process. Moreover, the KEGG analysis results showed that the FmRNAs targeted by miR-130b were related to bacterial invasion of epithelial cells, Hippo signaling pathway, axon guidance, microRNAs in cancer, transcriptional misregulation in cancer, endocytosis, starch and sucrose metabolism, and so on.

### The Expression and Prognostic Value of the Hub FmRNAs Targeted by miR-130b in PC Tissues

We demonstrated the aberrant expression of the hub FmRNAs targeted by miR-130b (MET, PPARG, TBL1XR1, and TCF4) in PC using the GEPIA. Based on the optimal cut-off value with | Log_2_FC |> 1 and* P* < 0.01, the above four hub FmRNAs were all up-regulated in PC tissues, as demonstrated in Fig. 3d–g.

To study the correlation between the expression of related molecules and the prognosis of PC patients, survival analysis was performed on each FmRNA targeted by miR-130b in the miRNA-mRNA networks, and potential prognostic biomarkers in PC were found, as presented in Fig. 3h–l. The results showed that five FmRNAs, including MET, RACGAP1, SLC44A1, TBL1XR1, and TRPS1, seemed to have a protective effect, and the higher the expression level of these mRNAs, the better the prognosis of PC patients. Among them, only MET and TBL1XR1 were detected as hub mRNAs.

### miR-130b Decreased the Expression of MET and TBL1XR1 in PC

**Fig. 4:**
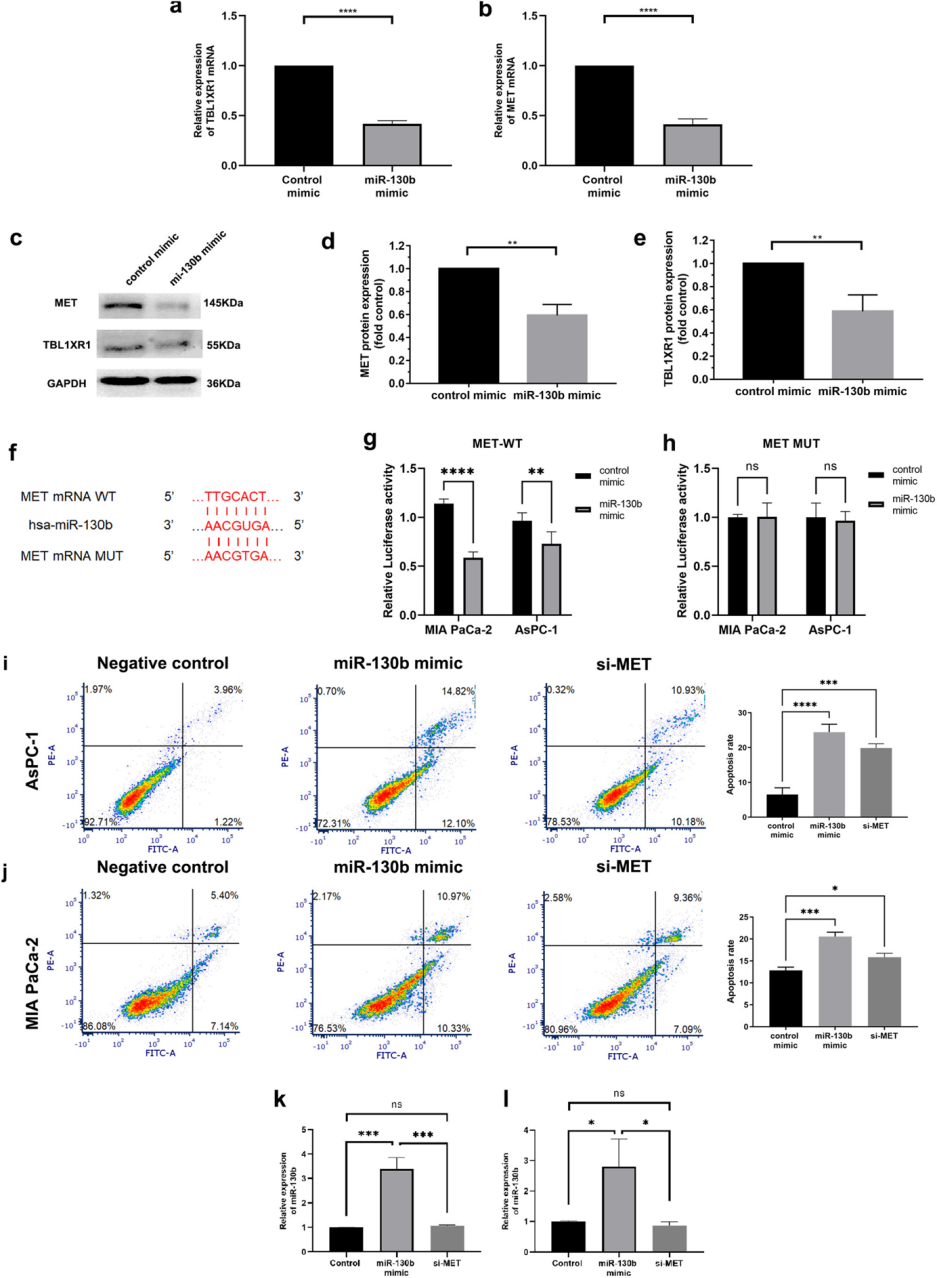
miR-130b overexpression inhibited the expression of MET and TBL1XR1 in PC cells, and could promote the apoptosis of PC cells by targeting MET. **a**, **b** The expression of TBL1XR1and MET after transfection with miR-130b mimic and control determined by RT-qPCR. **c–e** The expression of MET and TBL1XR1 after transfection with miR-130b mimic and control determined by western blotting. **f** Direct binding sequences of miR-130b and MET mRNA. **g, h** Luciferase activities of 3′UTR MET-luc constructs in PC cells after transfection with miR-130b mimic or control.** i** After AsPC-1 cells were infected with control, miR-130b mimic or si-MET, apoptosis rates were tested with flow cytometry experiments. The apoptosis proportion was obviously increased in AsPC-1 cells with miR-130b overexpression and MET inhibition. **j** After MIA PaCa-2 cells were infected with control, miR-130b mimic or si-MET, apoptosis rates were tested with flow cytometry experiments. The apoptosis proportion was increased in MIA PaCa-2 cells with miR-130b overexpression and MET inhibition. Apoptosis was represented by a percentage (*Q*2 + *Q*3)/(*Q*1 + *Q*2 + *Q*3 + *Q*4). Three replicates were performed for this experiment. The results were analysed with *t* tests (unpaired). **k, l** RT-qPCR assay was performed to detect the expression of miR-130b (**k**) and MET (**l**) after transfection with miR-130b mimic, si-MET, and controls in PC cells

In these two hub FmRNAs selected, MET can phosphorylate intracellular proteins and involve in apoptosis. According to past researches, TBL1XR1 is linked to tumour development, metastasis, chemoresistance, and subpar overall survival in a number of malignancies. These hub genes were associated with cell expansion, migration, apoptosis and other biological progress. However, just few studies were focus on the association between these PC hub genes and miR-130b. To detect the relationship, we analysed the expressions of MET and TBL1XR1 in AsPC-1 and MIA PaCa-2 cells transfected with miR-130b mimic or NC mimic. Notably, the expression of MET and TBL1XR1 were reduced in miR-130b overexpression groups compared with control groups both in RNA and protein levels (Fig. 4a–e). These results revealed miR-130b may mediate the expression of MET and TBL1XR1 in PC cells.

Then we performed KEGG analysis on MET and TBL1XR1. The result showed that TBL1XR1 was related to Wnt signaling pathway. On the other hand, MET was annotated to participate in 23 KEGG pathways, such as EGFR tyrosine kinase inhibitor resistance, MAPK, Ras, Rap1, Calcium, and PI3K-Akt signaling pathway, and so on. Based on above data, we investigated whether miR-130b regulated the malignant progress of PC cells, such as cell apoptosis, proliferation, migration or some special signaling pathway through modulating MET expression. Therefore, we further verified that miR-130b and MET had a direct binding site using TargetScan (Fig. 4f) and employed a dual-luciferase reporter gene assay to identify that miR-130b could bind to MET. The results showed that for MET wild-type plasmid transfection, unregulated miR-130b brought down the luciferase activity in PC cells (Fig. 4 g, h).

### miR-130b Promoted Apoptosis in PC Cells by Targeting MET

We assessed the apoptosis viability of PC cells transfected with the miR-130b mimic group, si-MET group, and the negative control group via flow cytometry. It revealed that high miR-130b and low MET content promoted apoptosis in PC cells compared with the negative control (Fig. 4i-j). The results suggested that miR-130b could promote apoptosis by mediating MET expression in PC cells.

### miR-130b Impeded Proliferation by Targeting MET in PC Cells

**Fig. 5:**
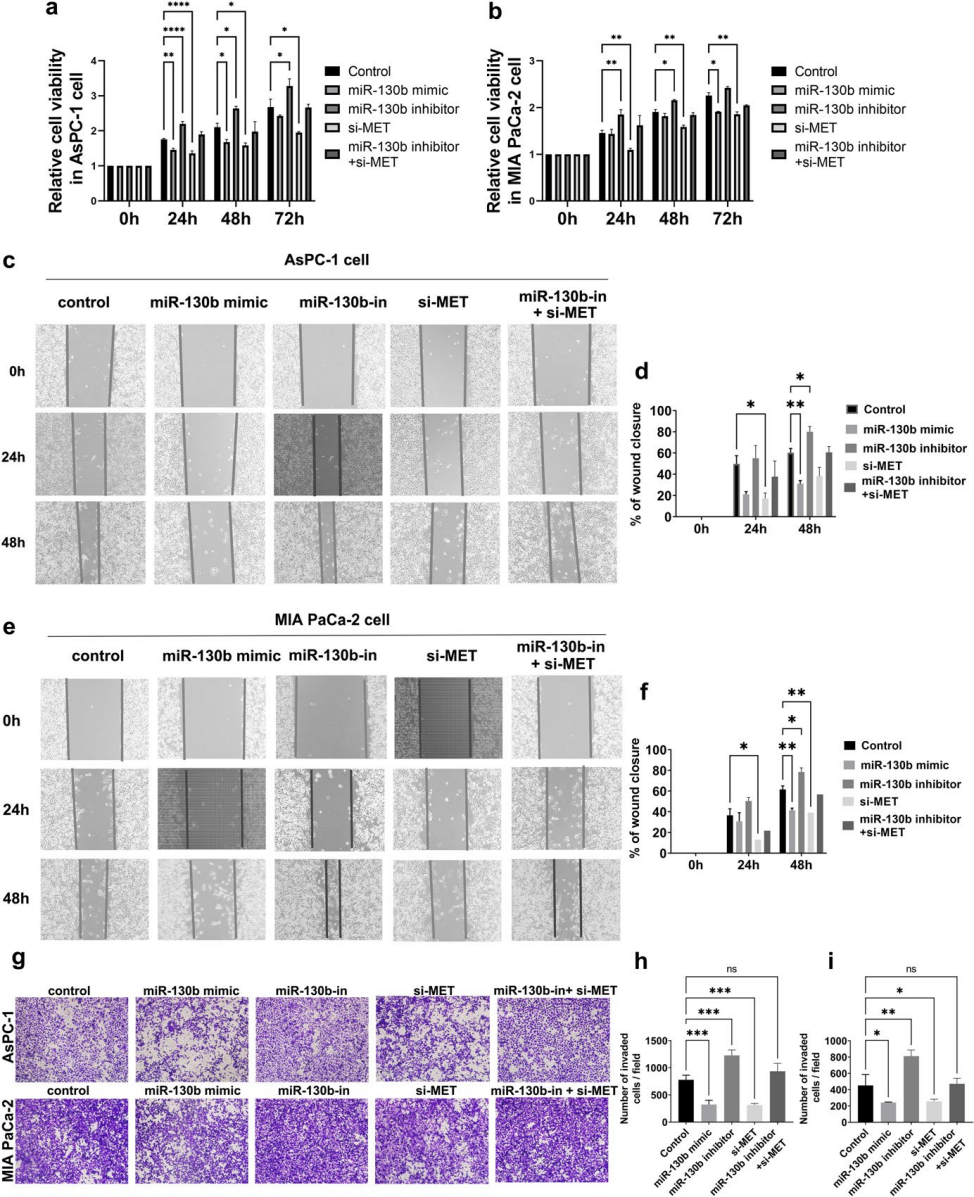
miR-130b suppressed cell proliferation and migration ability in PC by targeting MET. **a,b** CCK-8 assay was performed at different time points to detect the cell proliferation function of AsPC-1 cells (**a**) and MIA PaCa-2 cells (**b**), which transfected with miR-130b mimic, miR-130b inhibitor, si-MET, 130b inhibitor + si-MET, and control groups, respectively. All experiments were carried out in five replicates, and *t* tests (unpaired) were used to evaluate the results. **c, d** Cell mobilities of AsPC-1 cells transfected with miR-130b mimic, miR-130b inhibitor, si-MET, 130b inhibitor + si-MET, and control groups, respectively, were determined by wound healing assay at 0, 24, and 48 h after the scratching. **e–f** Cell mobilities of MIA PaCa-2 cells transfected with miR-130b mimic, miR-130b inhibitor, si-MET, 130b inhibitor + si-MET, and control groups, were determined by wound healing assay at 0, 24, 48 h after the scratching. **g-i** The migration ability of AsPC-1 cells and MIA PaCa-2 cells were assessed by transwell assay after transfection with miR-130b mimic, miR-130b inhibitor, si-MET, 130b inhibitor + si-MET, and control groups for 48 h. Three replications were performed for each experiment. *PC* pancreatic cancer, *CCK-8* cell counting kit-8; *P < 0.05, **P < 0.01, ***P < 0.001, and ****P < 0.00001 were considered statistically significant. *ns* no significant

To examine the modulation of miR-130b on proliferation activity of PC cells, we transfected miR-130b mimic, miR-130b inhibitor, si-MET, miR-130b inhibitor + si-MET and control group into AsPC-1 cells, respectively. The proliferation rates of these five groups were then assessed at 0, 24, 48, and 72 h after transfection (Fig. 5a). The sequential proliferation rates indicated that miR-130b overexpression and MET inhibition inhibited the proliferation capacity of AsPC-1 cells and miR-130b inhibition increased the PC cell proliferation. The co-transfection of si-MET could significantly decrease the cell proliferation of miR-130b inhibitor group in comparison to miR-130b inhibitor group. Similar results were also observed in MIA PaCa-2 cells, as presented in Fig. 5b. Thus, the present findings indicated that miR-130b partially restrained PC cell proliferation via regulating MET expression.

### miR-130b Inhibited Migration by Targeting MET in PC Cells

The effects of miR-130b on the migration ability of PC cells were detected by wound-healing assay and transwell assay. In the wound-healing assay, compared to the beginning, AsPC-1 cells transfected with miR-130b mimic and si-MET had reduced mobility across single cell layers at 24 and 48 h after scratch, while the group transfected with miR-130b inhibitor increased the mobility across single cell layers. In addition, the co-transfection of si-MET could significantly decrease the cell proliferation in miR-130b inhibitor group compared with miR-130b inhibitor group, as shown in Fig. 5c, d. In MIA PaCa-2 cells, similar outcomes were also seen, as shown in Fig. 5e–f. In the transwell assays, miR-130b overexpression and MET down-expression significantly decreased cell migration abilities at 24 and 48 h post-seeding compared with the control group in AsPC-1 cells, while the inhibition of miR-130b resulted in increased cell migration (*P* < 0.05). The co-transfection of si-MET could significantly decrease the cell migration of miR-130b inhibitor group in comparison to that in the miR-130b inhibitor group, as shown in Fig. 5g, h. Similar outcomes were also observed in MIA PaCa-2 cells, as presented in Fig. 5g and 5i. Through wound-healing and transwell assay, we found that cell migration were promoted by miR-130b inhibitor, inhibited by miR-130b mimic and si-MET, and could be rescued by si-MET co-transfection in comparison to miR-130b inhibitor group. These evidences turned out that miR-130b restrained PC cell migration via regulating MET expression.

### miR-130b Inhibited the PI3K/Akt Signaling Pathway in PC Cells by Targeting MET

**Fig. 6:**
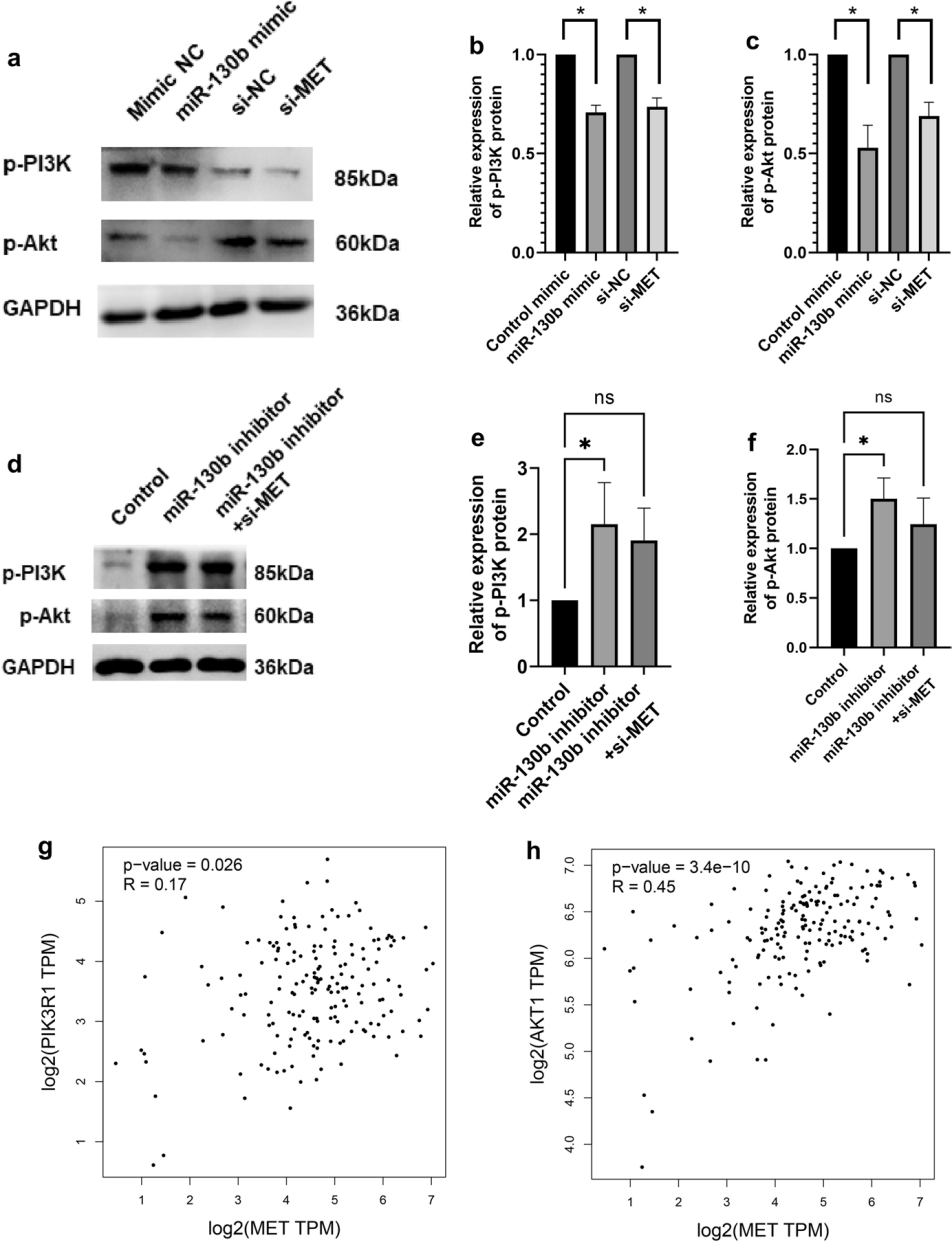
miR-130b impeded the PI3K/Akt signaling pathway by targeting MET in PC cells. **a–c** The expression levels of p-PI3K and p-Akt were analyzed by western blotting following 48-h of transfection with miR-130b mimic, si-MET and relative controls in PC cells; **d–f** The expression levels of p-PI3K and p-Akt were analyzed by western blotting following 48-h of transfection with miR-130b inhibitor, miR-130b inhibitor + si-MET and relative controls in PC cells; **g** The spearson analysis of MET and PIK3R1 using GEPIA; **h** The spearson analysis of between MET and AKT1 using GEPIA. *P < 0.05 was considered statistically significant. *ns* no significant

It has been reported that MET is a substrate, and is a crucial protein in regulating cell apoptosis and signaling. According to the KEGG analysis, MET participated in the PI3K/Akt signaling pathway (Online Resource 6). To further investigate whether miR-130b modulated PI3K/Akt signaling pathway via targeting MET, we verified the expression of PI3K/Akt pathway-associated proteins by western blot. The results showed that the expression of p- PI3K and p-Akt were both decreased significantly in the miR-130b overexpression and si-MET group (Fig. 6a–c). The rescue experiment showed that the expression of p- PI3K and p-Akt were both increased in the miR-130b inhibitor group, and the co-transfection of si-MET could significantly decrease the expression of p- PI3K and p-Akt protein in miR-130b inhibitor group in comparison to that in the miR-130b inhibitor group (Fig. 6d, e). In addition, we used GEPIA to investigate the relationship between MET and genes encoded proteins associated with signaling pathways, such as PIK3R1 and AKT1. The results verified that MET was positively correlated with AKT1 and PIK3R1 (Fig. 6f, g). These findings suggested that miR-130b could mediated the PI3K/Akt pathway in PC cells by targeting MET.

## Discussion

PC is widely known for its high mortality and poor prognosis, which encourages us to explore more effective treatment measures and identify new prognostic indicators to improve the current situation. More and more studies have verified that abnormally expressed miRNAs and their target genes are significant biomarkers or regulators in many diseases, including PC (Fathi et al. 2021; Zhou et al. 2021). The construction of miRNA–mRNA regulatory networks will enable deeper acknowledgement of the molecular mechanisms underlying PC. As a result, identifying the irregularly expressed miRNAs and target genes linked to PC is crucial for improving patient prognosis.

In the study, we identified 40 DEmiRNAs and 1613 DEmRNAs from 365 PC tissues and 70 non-tumour tissues obtained from six GEO datasets. We then identified seven hub DEmiRNAs and 10 hub FmRNAs by constructing the miRNA-mRNA regulatory networks and the PPI network. The results demonstrated that miR-130b was the key miRNA with the highest degrees in the miRNA-mRNA networks. For miR-130b, several previous studies have explored its effect in different types of cancer. In some cancers, such as medulloblastoma (MB) (Huang et al. 2020), prostate cancer (Mu et al. 2020), and glioma (Gu et al. 2018), miR-130b was considered to be a cancer suppressor. On the other hand, miR-130b was considered to be a cancer-promoter in human hepatocellular carcinoma (Ou et al. 2020), oral squamous cell carcinoma (W. Yan et al. 2021), and esophageal squamous cell carcinoma (Liu et al. 2021). In our study, miR-130b expression was lower in PC tissues than in non-carcinoma samples in multiple sequencing datasets selected from the GEO database, as well as in RT-qPCR assay. After analysing the RT-qPCR data, we found that the prognosis of high miR-130b subgroup was different with the low miR-130b subgroup, which was consistent with the finding of some earlier studies. Taking together, miR-130b had the potential to be a biomarker for cancer surveillance.

To date, more attention has been paid to validating the expression of miRNAs and their molecular effects in PC. Accordingly, we used multiple bioinformatics integration tools to verify the intrinsic genetic interactive relationship of miR-130b and its downstream target genes. We detected 17 FmRNAs targeted by miR-130b, and built a PPI network containing 69 FmRNAs which projected 10 genes (ALDH6A1, FN1, PPARG, MET, TCF4, ZEB1, CDH11, TBL1XR1, VCAN, and PLEK) as hub genes in PC from 6 GEO datasets. MET and TBL1XR1, targeted by miR-130b, were associated with the survival of PC patients. In our study, MET and TBL1XR1 may play important roles in PC progression, and we indicated that MET was the direct downstream target of miR-130b confirmed by luciferase assay. An increasing number of studies verified that proto-oncogene MET was involved in the initiation and development of PC (Li et al. 2021; Escorcia et al. 2020; Shi et al. 2022). Modica et al*.* showed that the HGF/MET axis involved tenascin-C tumour secretion and facilitated stromal rewiring in PC (Modica et al. 2021). MET inhibitors may increase the chemotherapeutic treatment resistance of PC patients (Firuzi et al. 2019). Furthermore, Cannistraci et al*.* demonstrated that Met/miR-130b axis might be regarded as a new prognostic indicator for assessing risk in patients and an indicator of treatment resistance in prostate tumour (Cannistraci et al. 2017). Therefore, we further explored the interaction between miR-130b and MET in PC cells and revealed that miR-130b overexpression could affect MET expression at both the RNA and protein levels. In conclusion, miR-130b may affect the occurrence and development of PC by targeting MET.

We conducted several cell studies to show the molecular roles of miR-130b and MET in PC. The results showed that miR-130b could promote cell apoptosis, suppress proliferation and migration abilities in PC cells by targeting MET. We also observed the correlation relevance between miR-130b and PC at the cellular level. To sum up, miR-130b and MET expression levels that were abnormal may be a predictive factor of PC prognosis and may be linked to PC formation and progression.

Therefore, bioinformatics analyses and cell function experiments were performed to clarify the roles of miR-130b on PC, to find out and confirm related downstream targets, and provide a basis for comprehensive research. Using KEGG analysis, TBL1XR1 was discovered to be associated with the Wnt signaling pathway, and MET gene was associated with 23 KEGG pathways including PI3K/Akt signaling pathway. Based on these, we further investigated whether miR-130b modulated PI3K/Akt signaling pathway via targeting MET. The results showed that the PI3K/Akt pathway was inhibited by miR-130b overexpression and MET downregulation, and activated by miR-130b inhibition. It suggested that the miR-130b may impede PI3K/Akt signaling pathway by targeting MET. Our results were similar to those of Chen et al. (2022), Fukuhisa et al. (2019), and Zhao et al. (2013). Chen P believed that miR-130b could regulate RHOA and exhibit tumor suppressor functions in PC by targeting RHOA (Chen et al. 2022). Fukuhisa H demonstrated that antitumor downregulated miR-130b-5p may enhance the proliferation, migration, and invasion of cancer cells by mediating EPS8 (Fukuhisa et al. 2019). Zhao G explored that miR-130b could suppress cell growth and invasion abilities in PC by acting on STAT3 (Zhao et al. 2013). In our study, we have adopted more and more comprehensive integrated bioinformatics analysis, and completed cell function tests, and explored the relationship and KEGG signaling pathways mediated by potential targets and miR-130b. Based on these, we suggested that miR-130b and MET may be potential anti-tumour targets for new treatment of PC.

In conclusion, this study confirmed that miR-130b was lowly expressed in PC and inhibited the proliferation and migration ability, promote cell apoptosis, and inhibit PI3K/Akt signaling pathway by targeting MET. These results from integrated analysis may provide new ideas for studying physiopathological mechanisms.

## Conclusions

In brief, we identified that miR-130b was a significantly downregulated miRNA in PC, and MET was a key target of miR-130b through integrated analysis. miR-130b suppressed the proliferation and migration, promoted cell apoptosis and suppressed the PI3K/Akt signaling pathway by targeting MET in PC. These results suggested that miR-130b and MET may provide new ideas as anti-tumour targets or prognostic indicators of PC.

## Supplementary Information

Below is the link to the electronic supplementary material.

Supplementary file1 (XLSX 10 KB)

Supplementary file2 (XLSX 9 KB)

Supplementary file3 (XLSX 115 KB)

Supplementary file4 (XLSX 11 KB)

Supplementary file5 (XLSX 9 KB)

Supplementary file6 (XLSX 10 KB)

## Data Availability

Publicly available datasets were used in this study. These can be found in the GEO database at https://www.ncbi.nlm.nih.gov/geo. In addition to the publicly available datasets, all data on the manuscripts that support the findings of this study are included within this paper and its Supplementary Information files. The authors declare that there is no conflict of interest regarding the publication of this paper.

## Abbreviations

PC: Pancreatic cancer
miRNA: MicroRNA
mRNA: Messenger RNA
GEO: Gene Expression Omnibus
NC: Non-cancerous
DERNAs: Differentially expressed RNAs
FC: Fold change
GO: Gene Ontology
KEGG: Kyoto Encyclopedia of Genes and Genomes
Webgestalt: WEB-based GEne SeT AnaLysis Toolkit
MF: Molecular function
CC: Cellular component
BP: Biological process
FmRNAs: Final functional mRNAs
PPI: Protein–protein interaction
STRING: Search Tool for the Retrieval of Interacting Genes database
RT-qPCR: Reverse transcription quantitative polymerase chain reaction assays
GEPIA: Gene Expression Profiling Interactive Analysis database
TCGA: The Cancer Genome Atlas
GTEx: Genotype Tissue Expression
DMEM: Dulbecco’s modified Eagle’s medium
RIPA: Radioimmunoprecipitation
PMSF: Phenylmethylsulfonyl fluoride
CCK-8: Cell counting kit-8
PI: Propidium Iodide
CEA: Carcinoembryonic antigen
CA19-9: Carbohydrate antigen 19-9
MB: Medulloblastoma

## References

[CR1] Agarwal V, Bell GW, Nam JW, Bartel DP (2015) Predicting effective microRNA target sites in mammalian mRNAs. Elife. 10.7554/eLife.05005 26267216 10.7554/eLife.05005 PMC4532895

[CR2] Barrett T, Wilhite SE, Ledoux P, Evangelista C, Kim IF, Tomashevsky M et al (2013) NCBI GEO: archive for functional genomics data sets–update. Nucleic Acids Res 41(Database Issue):D991–D995. 10.1093/nar/gks1193 23193258 10.1093/nar/gks1193 PMC3531084

[CR3] Bauer AS, Keller A, Costello E, Greenhalf W, Bier M, Borries A et al (2012) Diagnosis of pancreatic ductal adenocarcinoma and chronic pancreatitis by measurement of microRNA abundance in blood and tissue. PLoS ONE 7(4):e34151. 10.1371/journal.pone.0034151 22511932 10.1371/journal.pone.0034151 PMC3325244

[CR4] Bi H, Fei Q, Li R, Liu B, Xia R, Char SN et al (2020) Disruption of miRNA sequences by TALENs and CRISPR/Cas9 induces varied lengths of miRNA production. Plant Biotechnol J 18(7):1526–1536. 10.1111/pbi.13315 31821678 10.1111/pbi.13315 PMC7292542

[CR5] Braga L, Ali H, Secco I, Giacca M (2021) Non-coding RNA therapeutics for cardiac regeneration. Cardiovasc Res 117(3):674–693. 10.1093/cvr/cvaa071 32215566 10.1093/cvr/cvaa071 PMC7898953

[CR6] Cannistraci A, Federici G, Addario A, Di Pace AL, Grassi L, Muto G et al (2017) C-Met/miR-130b axis as novel mechanism and biomarker for castration resistance state acquisition. Oncogene 36(26):3718–3728. 10.1038/onc.2016.505 28192399 10.1038/onc.2016.505

[CR7] Chen Y, Wang X (2020) miRDB: an online database for prediction of functional microRNA targets. Nucleic Acids Res 48(D1):D127–D131. 10.1093/nar/gkz757 31504780 10.1093/nar/gkz757 PMC6943051

[CR8] Chen P, Zeng Z, Wang J, Cao W, Song C, Lei S et al (2022) Long noncoding RNA LINC00857 promotes pancreatic cancer proliferation and metastasis by regulating the miR-130b/RHOA axis. Cell Death Discov 8(1):198. 10.1038/s41420-022-01008-2 35418193 10.1038/s41420-022-01008-2 PMC9008000

[CR9] Diboun I, Wernisch L, Orengo CA, Koltzenburg M (2006) Microarray analysis after RNA amplification can detect pronounced differences in gene expression using limma. BMC Genomics 7:252. 10.1186/1471-2164-7-252 17029630 10.1186/1471-2164-7-252 PMC1618401

[CR10] Donahue TR, Tran LM, Hill R, Li Y, Kovochich A, Calvopina JH et al (2012) Integrative survival-based molecular profiling of human pancreatic cancer. Clin Cancer Res 18(5):1352–1363. 10.1158/1078-0432.CCR-11-1539 22261810 10.1158/1078-0432.CCR-11-1539 PMC3816537

[CR11] Escorcia FE, Houghton JL, Abdel-Atti D, Pereira PR, Cho A, Gutsche NT et al (2020) ImmunoPET predicts response to met-targeted radioligand therapy in models of pancreatic cancer resistant to met kinase inhibitors. Theranostics 10(1):151–165. 10.7150/thno.37098 31903112 10.7150/thno.37098 PMC6929627

[CR12] Fathi M, Ghafouri-Fard S, Abak A, Taheri M (2021) Emerging roles of miRNAs in the development of pancreatic cancer. Biomed Pharmacother 141:111914. 10.1016/j.biopha.2021.111914 34328099 10.1016/j.biopha.2021.111914

[CR13] Firuzi O, Che PP, El Hassouni B, Buijs M, Coppola S, Lohr M et al (2019) Role of c-MET inhibitors in overcoming drug resistance in spheroid models of primary human pancreatic cancer and stellate cells. Cancers (Basel). 10.3390/cancers11050638 31072019 10.3390/cancers11050638 PMC6562408

[CR14] Frampton AE, Castellano L, Colombo T, Giovannetti E, Krell J, Jacob J et al (2014) MicroRNAs cooperatively inhibit a network of tumor suppressor genes to promote pancreatic tumor growth and progression. Gastroenterology 146(1):268-277.e18. 10.1053/j.gastro.2013.10.010 24120476 10.1053/j.gastro.2013.10.010

[CR15] Fukuhisa H, Seki N, Idichi T, Kurahara H, Yamada Y, Toda H et al (2019) Gene regulation by antitumor miR-130b-5p in pancreatic ductal adenocarcinoma: the clinical significance of oncogenic EPS8. J Hum Genet 64(6):521–534. 10.1038/s10038-019-0584-6 30858505 10.1038/s10038-019-0584-6

[CR16] Gu JJ, Fan KC, Zhang JH, Chen HJ, Wang SS (2018) Suppression of microRNA-130b inhibits glioma cell proliferation and invasion, and induces apoptosis by PTEN/AKT signaling. Int J Mol Med 41(1):284–292. 10.3892/ijmm.2017.3233 29115407 10.3892/ijmm.2017.3233 PMC5746316

[CR17] Gu J, Xu H, Chen Y, Li N, Hou X (2022) MiR-223 as a regulator and therapeutic target in liver diseases. Front Immunol 13:860661. 10.3389/fimmu.2022.860661 35371024 10.3389/fimmu.2022.860661 PMC8965842

[CR18] Haider S, Wang J, Nagano A, Desai A, Arumugam P, Dumartin L et al (2014) A multi-gene signature predicts outcome in patients with pancreatic ductal adenocarcinoma. Genome Med 6(12):105. 10.1186/s13073-014-0105-3 25587357 10.1186/s13073-014-0105-3 PMC4293116

[CR19] Hirabayashi K, Miyazawa M, Takanashi Y, Morimachi M, Kawanishi A, Saika T et al (2022) miR-4653-3p overexpression is associated with a poor prognosis of pancreatic ductal adenocarcinoma via HIPK2 downregulation. Sci Rep 12(1):17927. 10.1038/s41598-022-22950-2 36289359 10.1038/s41598-022-22950-2 PMC9606280

[CR20] Huang S, Xue P, Han X, Zhang C, Yang L, Liu L et al (2020) Exosomal miR-130b-3p targets SIK1 to inhibit medulloblastoma tumorigenesis. Cell Death Dis 11(6):408. 10.1038/s41419-020-2621-y 32483145 10.1038/s41419-020-2621-y PMC7264172

[CR21] Janky R, Binda MM, Allemeersch J, Van den Broeck A, Govaere O, Swinnen JV et al (2016) Prognostic relevance of molecular subtypes and master regulators in pancreatic ductal adenocarcinoma. BMC Cancer 16:632. 10.1186/s12885-016-2540-6 27520560 10.1186/s12885-016-2540-6 PMC4983037

[CR22] Li E, Huang X, Zhang G, Liang T (2021) Combinational blockade of MET and PD-L1 improves pancreatic cancer immunotherapeutic efficacy. J Exp Clin Cancer Res 40(1):279. 10.1186/s13046-021-02055-w 34479614 10.1186/s13046-021-02055-w PMC8414725

[CR23] Li M, Zhou J, Zhang Z, Li J, Wang F, Ma L et al (2022) Exosomal miR-485-3p derived from pancreatic ductal epithelial cells inhibits pancreatic cancer metastasis through targeting PAK1. Chin Med J (Engl) 135(19):2326–2337. 10.1097/CM9.0000000000002154 36535010 10.1097/CM9.0000000000002154 PMC9771326

[CR24] Liao Y, Wang J, Jaehnig EJ, Shi Z, Zhang B (2019) WebGestalt 2019: gene set analysis toolkit with revamped UIs and APIs. Nucleic Acids Res 47(W1):W199–W205. 10.1093/nar/gkz401 31114916 10.1093/nar/gkz401 PMC6602449

[CR25] Liu J, Zhou R, Deng M, Xue N, Li T, Guo Y et al (2021) Long non-coding RNA DIO3OS binds to microRNA-130b to restore radiosensitivity in esophageal squamous cell carcinoma by upregulating PAX9. Cancer Gene Ther. 10.1038/s41417-021-00344-2 34183777 10.1038/s41417-021-00344-2

[CR26] Mizrahi JD, Surana R, Valle JW, Shroff RT (2020) Pancreatic cancer. Lancet 395(10242):2008–2020. 10.1016/S0140-6736(20)30974-0 32593337 10.1016/S0140-6736(20)30974-0

[CR27] Modica C, Olivero M, Zuppini F, Milan M, Basilico C, Vigna E (2021) HGF/MET axis induces tumor secretion of tenascin-c and promotes stromal rewiring in pancreatic cancer. Cancers (Basel). 10.3390/cancers13143519 34298732 10.3390/cancers13143519 PMC8305254

[CR28] Mu HQ, He YH, Wang SB, Yang S, Wang YJ, Nan CJ et al (2020) MiR-130b/TNF-alpha/NF-kappaB/VEGFA loop inhibits prostate cancer angiogenesis. Clin Transl Oncol 22(1):111–121. 10.1007/s12094-019-02217-5 31667686 10.1007/s12094-019-02217-5

[CR29] Ou C, Peng NF, Li H, Peng YC, Li LQ (2020) The potential mechanism of miR-130b on promotion of the invasion and metastasis of hepatocellular carcinoma by inhibiting Notch-Dll1. J Recept Signal Transduct Res 40(2):157–165. 10.1080/10799893.2020.1721537 32019397 10.1080/10799893.2020.1721537

[CR30] Sandhu V, Bowitz Lothe IM, Labori KJ, Lingjaerde OC, Buanes T, Dalsgaard AM et al (2015) Molecular signatures of mRNAs and miRNAs as prognostic biomarkers in pancreatobiliary and intestinal types of periampullary adenocarcinomas. Mol Oncol 9(4):758–771. 10.1016/j.molonc.2014.12.002 25579086 10.1016/j.molonc.2014.12.002 PMC5528780

[CR31] Shi X, Wang M, Zhang Y, Guo X, Liu M, Zhou Z et al (2022) Hypoxia activated HGF expression in pancreatic stellate cells confers resistance of pancreatic cancer cells to EGFR inhibition. EBioMedicine 86:104352. 10.1016/j.ebiom.2022.104352 36371988 10.1016/j.ebiom.2022.104352 PMC9664470

[CR32] Sticht C, De La Torre C, Parveen A, Gretz N (2018) miRWalk: an online resource for prediction of microRNA binding sites. PLoS ONE 13(10):e0206239. 10.1371/journal.pone.0206239 30335862 10.1371/journal.pone.0206239 PMC6193719

[CR33] Strobel O, Neoptolemos J, Jager D, Buchler MW (2019) Optimizing the outcomes of pancreatic cancer surgery. Nat Rev Clin Oncol 16(1):11–26. 10.1038/s41571-018-0112-1 30341417 10.1038/s41571-018-0112-1

[CR34] Tang Z, Li C, Kang B, Gao G, Li C, Zhang Z (2017) GEPIA: a web server for cancer and normal gene expression profiling and interactive analyses. Nucleic Acids Res 45(W1):W98–W102. 10.1093/nar/gkx247 28407145 10.1093/nar/gkx247 PMC5570223

[CR35] Vasaikar SV, Straub P, Wang J, Zhang B (2018) LinkedOmics: analyzing multi-omics data within and across 32 cancer types. Nucleic Acids Res 46(D1):D956–D963. 10.1093/nar/gkx1090 29136207 10.1093/nar/gkx1090 PMC5753188

[CR36] Yan S, Wang H, Chen X, Liang C, Shang W, Wang L et al (2020) MiR-182-5p inhibits colon cancer tumorigenesis, angiogenesis, and lymphangiogenesis by directly downregulating VEGF-C. Cancer Lett 488:18–26. 10.1016/j.canlet.2020.04.021 32473243 10.1016/j.canlet.2020.04.021

[CR37] Yan W, Wang Y, Chen Y, Guo Y, Li Q, Wei X (2021) Exosomal miR-130b-3p promotes progression and tubular formation through targeting PTEN in oral squamous cell carcinoma. Front Cell Dev Biol 9:616306. 10.3389/fcell.2021.616306 33829013 10.3389/fcell.2021.616306 PMC8019696

[CR38] Yang Z, Tang Y, Wu X, Wang J, Yao W (2022) Construction and investigation of an lncRNA-miRNA-mRNA regulatory network of pancreatic cancer via bioinformatics analysis, PREPRINT (Version 1). Research Square. 10.21203/rs.3.rs-1433634/v1

[CR39] Yu Q, Xiu Z, Jian Y, Zhou J, Chen X, Chen X et al (2022) microRNA-497 prevents pancreatic cancer stem cell gemcitabine resistance, migration, and invasion by directly targeting nuclear factor kappa B1. Aging (Albany, NY) 14(14):5908–5924. 10.18632/aging.204193 35896012 10.18632/aging.204193 PMC9365558

[CR40] Zhao G, Zhang JG, Shi Y, Qin Q, Liu Y, Wang B et al (2013) MiR-130b is a prognostic marker and inhibits cell proliferation and invasion in pancreatic cancer through targeting STAT3. PLoS ONE 8(9):e73803. 10.1371/journal.pone.0073803 24040078 10.1371/journal.pone.0073803 PMC3769379

[CR41] Zhou Y, Zhu Y, Dong X, Cao G, Li Y, Fan Y et al (2021) Exosomes derived from pancreatic cancer cells induce osteoclast differentiation through the miR125a-5p/TNFRSF1B pathway. Onco Targets Ther 14:2727–2739. 10.2147/OTT.S282319 33907416 10.2147/OTT.S282319 PMC8064725

